# Bloodstream Infection and Gram-Negative Resistance: The Role for Newer Antibiotics

**DOI:** 10.3390/antibiotics12060977

**Published:** 2023-05-29

**Authors:** Jessica Waters, Andrew F. Shorr

**Affiliations:** Division of Pulmonary and Critical Care Medicine, Medstar Washington Hospital Center, Washington, DC 20010, USA; jessica.l.waters@medstar.net

**Keywords:** antibiotics, bacteremia, bloodstream infection, Gram-negative, resistance

## Abstract

Gram-negative resistance remains a major challenge. Rates of in vitro resistance to commonly utilized antibiotics have skyrocketed over the last decade. Clinicians now encounter multidrug-resistant organisms routinely. Fortunately, newer agents, such as ceftazidime–avibactam, ceftolozone–tazobactam, meropenem–vaborbactam, and cefiderocol, have been developed and are now available for use against these pathogens. Clinical trials with these novel therapies have focused on multiple infection types ranging from complicated urinary tract infections to nosocomial pneumonia. Nonetheless, there remains little information about the efficacy of these drugs for bacteremia. To better appreciate the types and limitations of the evidence supporting the role for these unique molecules in bloodstream infection, one requires an appreciation of the initial clinical trials supporting the regulatory approval of these antibiotics. Furthermore, physicians must understand the subsequent case series and reports specifically focusing on outcomes for patients with bacteremia treated with these drugs. Despite the limitations of the data and reports relating to treatment for bacteremia with these antibiotics, each agent appears to be efficacious and can provide good outcomes in bloodstream infections due to resistant pathogens.

## 1. Introduction

Approximately 250,000 bloodstream infections (BSIs) occur in the United States every year [1], including both those present on admission and ones which develop as a nosocomial infection. More importantly, BSIs remain a major cause of morbidity and mortality, irrespective of whether they represent primary infections or arise as secondary complications of infections elsewhere [2]. Several analyses, furthermore, suggest that BSIs confer an attributable mortality risk ranging from 7 to 35% [3,4]. Gram-negative rods (GNRs), while less common than Gram-positive cocci in BSIs, often underlie cases of sepsis [5].

Continuing trends in the evolution of antimicrobial resistance (AMR) add to the threat of and clinical challenge related to BSIs. Some estimate that 35,000 deaths occur annually in the US due to resistant organisms, while the global burden is even more staggering [6,7]. AMR can prove particularly dangerous in Gram-negative infections, where the prevalence of in vitro resistance to first-line antibiotics is growing at an alarming rate [8]. For multiple pathogens, standard initial treatments can no longer be reliably employed. This leads clinicians to reach for broader agents—which, in turn, only fuels the vicious cycle of AMR. At the bedside, these concerning rates of AMR increase the potential that the patient will receive inappropriate antibiotic therapy—a major risk factor for death [9]. Initial appropriate antimicrobial therapy essentially represents the only modifiable factor that can alter outcomes in severe infection. Delaying effective antibiotic therapy in septic shock, for example, raises mortality by 7% for every hour of delay [10]. In addition to its impact on length of stay, delays in delivering appropriate therapy also increase length of stay and hospital costs [11]. The costs of AMR via its direct impact on the potential for inappropriate therapy results in longer durations of hospitalizations and a greater chance for subsequent hospital readmission [11].

Fortunately, over the last 5–10 years, multiple novel agents have come to the bedside. Although distinct in their respective spectrums of activity, these novel therapies represent important additions to the clinician’s armamentarium. None of these novel agents have been specifically evaluated in BSI per se. Rather, the data describing their utility for GNR-resistant BSIs derive from subgroup analyses of large, randomized phase III trials conducted for regulatory approval and from various case series published following a new drug’s introduction into the hospital. More specifically, those treating BSIs due to resistant Gram-negative organisms need to appreciate the data for this syndrome as it relates to ceftazidime–avibactam (CAZ), ceftolozane–tazobactam (CT), meropenem–vaborbactam (MV), and cefiderocol.

In addition to understanding the clinical activity of these agents in BSIs, it is also important for readers to appreciate the differences between these agents in terms of their in vitro activity relative to select, important mechanisms of resistance. The Table A1 compares these drugs and highlights important distinctions between them.

Methodologically, we focused our narrative review initially by extracting data from large, randomized trials conducted for these agents as part of the regulatory approval process. Furthermore, we searched Medline to identify post-approval studies that contained data on patients suffering from BSIs and their relevant clinical outcomes.

## 2. Discussion

### 2.1. Ceftazidime–Avibactam

CAZ represents a combination of ceftazidime with avibactam, a non–β-lactam β-lactamase inhibitor that restores ceftazidime’s activity against Ambler class A, Class C, and some Class D β-lactamase-producing pathogens [12,13]. CAZ has been approved in the US for complicated intraabdominal infections (cIAI), complicated urinary tract infections (cUTI), and nosocomial pneumonia (NP) [14]. Of note, patients infected with carbapenem-resistant Enterobacteriaceae (CRE) isolates were excluded from these studies. However, CAZ is used to treat serious CRE infections given its activity against these pathogens [15,16].

In the RECLAIM 1 and 2 trials, investigators compared CAZ plus metronidazole to meropenem in patients with cIAIs (n = 1066) [17]. CAZ was found to be noninferior to meropenem. Across all primary analysis populations and in patients with ceftazidime-resistant GNR, CAZ combined with metronidazole exhibited similar clinical cure rates to meropenem. In RECLAIM 3, another randomized, double-blind study assessing the efficacy of CAZ plus metronidazole in cIAI, only 2.3% of the patients in the CAZ arm had a BSI along with their cIAI [18]. Outcomes in this small, bacteremic subgroup were similar to those seen in the overall population.

Similarly, REPROVE served as a multinational phase 3, double-blind noninferiority trial, which found CAZ to be noninferior to meropenem in patients with NP. In the study population, the predominant GNR pathogens included Klebsiella pneumoniae and Pseudomonas aeruginosa [19]. Only a small number of subjects had concurrent BSIs, as was the case with other studies with CAZ, thus limiting the applicability of this data for issues related to BSI treatment. In contrast, the REPRISE study represented a pathogen-directed rather than syndrome-focused clinical trial [20]. In this analysis, investigators compared CAZ to the best available therapy (BAT) in patients with cUTI or cIAI caused by ceftazidime-resistant Enterobacteriaceae or *P. aeruginosa*. In the BAT group, 97% of patients received a carbapenem. Regarding patients with bacteremia, 4% of patients with cUTI had bacteremia. None of the patients with cIAI had bacteremia. Patients with BSI had generally good outcomes.

Taking advantage of these multiple phase III reports, Mazuski et al. performed a post hoc pooled subset analysis of 101 bacteremic patients extracted from all 5 initial trials [21]. The clinical cure rates were 87.0% for CAZ and 83.0% for comparators. Generally, bacteremic patients analyzed in this subset were slightly older than in the overall population and more severely ill. The most commonly isolated organisms were *Enterobacteriaceae coli*, *P. aeruginosa*, *K. pneumoniae*, and *Enterobacter cloacae*. Approximately one third of these bacteremic patients had an infection with a ceftazidime-resistant organism, and 54.5% of those treated with CAZ demonstrated a favorable microbiological response vs. 47.4% of those treated with comparators.

In an important retrospective single-center study, Shields et al. evaluated 109 patients with CRE bacteremia who received CAZ versus therapy with either a carbapenem plus an aminoglycoside, a carbapenem plus colistin, or other another regimen including monotherapy with an aminoglycoside or colistin [22]. Crucially, these investigators noted a significantly higher rate of clinical success and survival for regimens containing CAZ. In logistic regression, receipt of CAZ was an independent predictor of clinical success. More importantly, survival at 30 and 90 days was 92% among patients receiving CAZ regimens versus 69% (carbapenem and aminoglycoside) and 55% (carbapenem and colistin) for various comparators. Readers should note that the patients in this analysis were severely ill, with many requiring care in an ICU. Although limited by its retrospective and single-center aspects, this study provides important data confirming the efficacy of CAZ in a population for which it was truly intended.

Hakeam et al. conducted a similar retrospective analysis of 61 adult patients with CRE bacteremia treated with CAZ or colistin [23]. Confirming the findings of Shields and colleagues, multivariate regression showed a lower 14-day mortality for patients treated with CAZ versus those given colistin. Despite an apparent early mortality benefit for CAZ, at thirty days no mortality differences were observed. Significantly, the majority of the cohort (88.5%) had secondary bacteremia, with the most commonly identified primary source being cIAI. The distributions of CRE isolates were similar between groups, and *K. pneumoniae* and *E. coli* were the most commonly implicated pathogens. Tumbarello et al. continued to build on these findings of others and subsequently evaluated patients treated for CRE (all *K. pneumoniae*) who received CAZ as salvage therapy [24]. Mortality at 30 days was significantly lower in patients treated with CAZ for bacteremia (36% compared with 55.8% for other antibiotics). Additionally, among bacteremic patients who were managed with single-drug salvage treatment regimens, CAZ recipients had improved survival.

Karaiskos and co-workers further helped to extend the knowledge of CAZ in bacteremia via a distinct, prospective, observational study of 147 adult patients [25]. In contrast to other case series, their population included organisms with OXA-48-producing pathogens and was prospective by design. After initial empiric therapy, patients received targeted therapy, consisting of either monotherapy with CAZ (46.3%) or CAZ in combination with at least another in vitro active agent. A cohort of 71 patients with a BSI due to CRE and who received treatment regimens that did not include CAZ (e.g., colistin, aminoglycosides, and tigecycline) served as the control population. Mortality was significantly lower with CAZ (13% vs. 40.8%, respectively). Furthermore, in multivariable analysis, only treatment with a CAZ-containing regimen represented an independent predictor of survival.

Information regarding CAZ for bacteremia is not restricted to studies in immunocompetent subjects. For example, in a small multicenter retrospective observational study of 31 adult patients with hematologic malignancies and CRE bacteremia, the efficacy of CAZ was assessed against other empiric treatment regimens consisting of carbapenems, aminoglycosides, tigecycline, fosfomycin, and colistin [26]. Most infections in this cohort were due to primary bacteremia (45.2%), which also makes it unique. Crude mortality in the CAZ group was numerically lower, though this difference was not statistically significant, likely due to the small sample size.

It is not surprising that, overall, the most information regarding treatment of resistant GNR BSIs exists for CAZ in comparison to other new antibiotics. CAZ was the first of the newer antibiotics approved and hence there has been more time to utilize and study the agent in the real world as opposed to clinical trial settings. Although prospective data for BSI therapy are limited, they at least exist—and this distinguishes it from other novel treatments. Readers should note that there have been issues with the emergence of resistance to CAZ [22,23,24]. Rates of emergence to resistance appear to vary across the globe [22,23,24]. Substitutions in beta-lactamase-related amino acids represent the main mechanism underlying resistance, although changes in efflux pumps have also been described.

### 2.2. Ceftolozane–Tazobactam

Ceftolozone–tazobactam (CT) combines a novel anti-pseudomonal cephalosporin with the well-known β-lactamase inhibitor tazobactam. It exhibits in vitro activity against extended-spectrum beta-lactamase (ESBL)-producing Enterobacteriaceae and drug-resistant *P. aeruginosa* [27]. In vitro studies have proven CT to be a potent antipseudomonal agent, active against multidrug-resistant strains [28]. It is approved as a result of multiple phase III clinical trials for the treatment of cUTIs, NP, and cIAIs [29,30,31].

ASPECT-cUTI, the initial phase III trial for CT, found this antibiotic to be superior to levofloxacin for cUTI [30]. The study included 1083 adult patients. The majority of cases had a monomicrobial infection with *E. coli* and, notably, 14.8% had an ESBL-producing organism. In the CT group, the composite cure rate was 76.9% vs. 68.4% in the levofloxacin comparator arm. Sustained clinical cure rates, though, were equivalent. Approximately 8% of patients were bacteremic at baseline, with most cases of bacteremia in patients diagnosed with pyelonephritis and caused by *E. coli*. A subgroup analysis of patients with BSI favored CT, with 79.3% achieving clinical cure vs. 57.6% of patients in the levofloxacin cohort. This difference, however, was not statistically significant.

ASPECT-NP addressed NP and was novel in that all patients were mechanically ventilated at baseline and that researchers employed a high-dose (3 g every 8 h) CT regimen [31]. CT performed well and met criteria for noninferiority with respect to mortality (the study’s primary endpoint). In contrast to many other trials in NP, the patients in this study were severely ill, with more than 40% receiving vasopressors during the study. In 31% of cases, ESBL-producing Enterobacteriaceae were isolated, and additionally, *P. aeruginosa* was a predominant pathogen. A small subpopulation in this trial, as was seen in the cUTI study, were bacteremic at baseline. In a subgroup analysis of these subjects, outcomes were statistically similar between CT and the comparator (meropenem). ASPECT cIAI was technically a phase II study but was large by phase II standards, enrolling nearly 1000 patients and examining intra-abdominal infections [29]. As generally seen with noninferiority designed trials, CT performed comparably to the control agent. In this study, as one might expect, few patients (<3%) were bacteremic.

Alternatively, the highest proportion of patients with a concurrent BSI in a regulatory study with CT came from a phase IV analysis of this agent in Japanese patients [32]. In an open-label study, 114 patients received CT for 7 days for cUTI, and more than a quarter of persons suffered from bacteremia. Illustrating the efficacy of CT, 22 of 23 bacteremic patients had negative blood cultures by the test-of-cure visit.

A number of subsequent reports support the utility of CT in GNR BSI. For example, Vena et al. evaluated the effect of CT for the treatment of severe drug-resistant *P. aeruginosa* infections [33]. In this retrospective report comparing CT to colistin and including both BSI and NP (n = 16), there was a trend towards a benefit with CT (81.3% vs. 56.3%). Although limited by its small sample size, these subjects were severely ill, and colistin served as the comparator—indicating that these subjects had few other alternatives available. Additionally, unlike other phase III reports, this analysis included some immunosuppressed subjects and thus underscores the potential efficacy of CT for BSI in a very complex population.

Khan et al. performed a similar retrospective review of adult patients with *P. aeruginosa* bacteremia that were non-susceptible to meropenem [34]. Of 40 cases, 11 patients (27.5%) received CT compared to 28 patients who received other agents. There was a significantly higher 30-day mortality in the CT group (54.5% vs. 13.8%). One key caveat, though, revolves around the fact that all isolates from the CT group were multidrug-resistant (MDR), while only 64.3% met criteria for MDR in the comparator group (*p* = 0.04). Readers should note that this study is further limited by its retrospective nature, small sample size, and potential selection bias—as indicated by the notable difference in rates of MDR prevalence. Most importantly, a conventional dosing strategy of 1.5 g of CT every 8 h was used—which may have been insufficient for *P. aeruginosa* bacteremia [35].

In an evaluation of real-world experience with CT in MDR *P. aeruginosa* BSI, Hakeam et al. reported outcomes in 40 subjects treated with various doses of CT or alternative agents [36]. The study included 46 subjects, with 17 receiving CT and 29 receiving a colistin-based regimen. Although there was no difference in mortality, which is not surprising given the sample size, these authors noted a higher clinical success rate with CT. Unlike other descriptions of outcomes in BSI, this review included persons suffering not only MDR infections but also individuals infected with extensively drug-resistant (XDR) strains of *P. aeruginosa*.

Finally, Bergas and colleagues evaluated CT in a heavily immunocompromised group of persons with hematologic malignancies and pseudomonal bacteremia—a highly lethal combination [37]. Utilizing a case control paradigm, they compared 44 cases treated with CT and 88 controls. The investigators matched the MDR profile of the isolate, though not precisely by the specific mechanism of resistance. Investigators found that the all-cause 30-day case fatality rate was significantly lower among the CT-treated subjects than in controls, 22.7% vs. 48.9%, respectively (*p* = 0.005). Furthermore, these researchers concluded that treatment with CT resulted in less need for mechanical ventilation (13.6% vs. 33.3%, *p* = 0.021).

Put simply, the data for relying on CT in MDR BSI seem robust in that the findings from subgroup reports deriving from phase III trials appear consistent with findings from various retrospective studies. Moreover, CT may afford a chance for BSI clearance and clinical cure in critically ill and immunosuppressed patients who often lack potential alternative treatment options—except for highly toxic, colistin-containing regimens.

### 2.3. Meropenem–Vaborbactam

Meropenem–vaborbactam (MV) combines meropenem with a novel, first in class, boron-based beta-lactamase inhibitor, vaborbactam [38]. Vaborbactam has potent activity against serine beta-lactamases [39]. In combining this drug with meropenem, it represents the first carbapenem and beta-lactamase inhibitor combination active against CRE. MV relies on a backbone of high-dose meropenem, and the combination is administered as a somewhat extended infusion [40]. The addition of the vaborbactam—in contrast to adding avibactam to ceftazidime—does not provide enhanced activity against *P. aeruginosa* [38,41]. In addition, unlike other agents we discuss, the approved indications for MV differ between the US and Europe. In the US, MV is approved for cUTI, while in Europe, it is also approved for use in NP [38].

As with other newer agents, the main trial exploring the efficacy of MV (Tango I) enrolled patients with cUTIs [42]. Tango I, though, relied upon piperacillin–tazobactam as the comparator (n = 550). Given the control agent, no patients with an MDR pathogen of interest were noted in this study. Additionally, only a small proportion of patients were bacteremic. Specifically, 12 patients in the MV group had bacteremia at baseline. Nonetheless, the overall success rate was 83.3% for those with a secondary BSI treated with MV.

Tango II, in contrast to Tango I, was an open label trial evaluating MV vs. BAT for CRE [43]. The study included 77 patients with confirmed or suspected CRE infections from a variety of potential sites. BAT ranged from polymyxins, carbapenems, aminoglycosides, and tigecycline to CAZ. Strikingly, cure rates were significantly higher in MV-treated patients (65.6% vs. 33.3%, *p* = 0.03). Readers should note that this trial was stopped early because of the suggestion of a survival benefit. Specifically, randomization to MV was associated with lower mortality (16% vs. 33%), though this difference was not statistically significant. In terms of those with a BSI, nearly 50% of the trial population were bacteremic—and in this sense, this trial represents a relatively large cohort of subjects with a BSI treated under the rubric of a randomized study. All-cause mortality rates in those with a BSI were numerically lower with MV: 28.6% vs. 37.5%.

In contrast to the situation surrounding CAZ and CT, there is a paucity of real-world reports investigating MV in BSI. However, Ackley et al. assessed MV in 131 adult patients who received MV vs. CAZ for treatment of a range of CRE infections, of which 40% included those with BSI [44]. These researchers observed similar rates of clinical success with either agent. Importantly, these authors report no episodes of resistance to MV developing on therapy.

In light of this limited experience with MV in bacteremia—whether in phase III trials or in real world, retrospective case series—clinicians may wish to proceed with caution when considering this agent for CRE bacteremia. The limited data become an even more acute issue for MV and BSI when considered in light of the multiple other agents with demonstrated efficacy in concurrent bacteremia.

### 2.4. Cefiderocol

Cefiderocol, the most recently approved of the agents we discuss, is a novel siderophore cephalosporin antibiotic with activity against many carbapenem-resistant bacteria, not just CRE [45]. It functions by binding to free iron and disrupting the cell wall of GNR. Once inside the periplasmic space, cefiderocol binds to penicillin-binding proteins (PBPs) [46]. Using a combination of this efficient cell entry along with its stability against beta-lactamase hydrolysis, cefiderocol is able to overcome the three main mechanisms of resistance observed in Gram-negative bacteria: enzymatic hydrolysis, porin channel mutation, and efflux pump overproduction [47]. As a consequence, cefiderocol provides in-vitro activity against most aerobic Gram-negative pathogens, both those susceptible and non-susceptible to carbapenems. Specifically, this agent has activity against ESBLs and CRE, *P. aeruginosa* and *Acinetobacter baumanii* [48].

Three randomized clinical trials have been conducted to evaluate the safety and efficacy of cefiderocol. APEKS-cUTI examined the efficacy of cefiderocol for cUTIs and compared cefiderocol to imipenem–cilastatin [49]. Cefiderocol was noninferior to imipenem–cilastatin for the primary endpoint of clinical cure and microbiologic eradication at the test-of-cure visit. Microbiological eradication rates were higher in the cefiderocol group. APEKS-NP focused on the efficacy of cefiderocol in treating NP caused by GNRs [50]. The trial compared cefiderocol to high-dose meropenem in 292 adult patients. Cefiderocol was again noninferior to meropenem in achieving the primary endpoint of all-cause mortality. Both APEKS-cUTI and APEKS-NP excluded patients with known carbapenem-resistant infections, though carbapenem resistance was found in some patients post randomization [49,50]. CREDIBLE-CR, on the other hand, addressed carbapenem-resistant GNRs [51]. The trial included patients with varying types of infections, including BSI, and found cefiderocol to have similar clinical and microbiological efficacy to BAT. However, the all-cause mortality rate was higher in the cefiderocol arm, mainly driven by outcomes among A. baumanii-infected subjects and those with pneumonia. Of the phase III regulatory studies exploring cefiderocol, only CREDIBLE-CR provided a subgroup analysis of patients with bacteremia. In this instance, cefiderocol appeared comparable to BAT regarding clinical cure and microbiological eradication in 37 patients with BSI and/or sepsis [51].

Paterson et al. conducted a post hoc analysis of outcomes in patients with bacteremia enrolled in the three regulatory studies for cefiderocol described above [52]. These investigators concluded that cefiderocol rapidly cleared bacteremia due to GNR pathogens. Across the populations included in the three regulatory studies, 84 of 885 (9.5%) randomized patients had positive baseline blood cultures, and 89 Gram-negative pathogens were isolated. Among Enterobacteriaceae species (n = 62), *E. coli* (n = 29) and K. pneumoniae (n = 23) were identified most frequently. Given the design of the various trials, nearly all of the carbapenem-resistant BSI isolates were derived from the CREDIBLE-CR report (34 of 37).

With respect to outcomes, on therapy, bacteremia eradication rates in the cefiderocol groups were 100% in APEKS-cUTI, 50% in APEKS-NP, and 72% in CREDIBLE-CR. This was similar to rates seen with the respective comparator agents for these studies (77.8%, 100%, and 69.2%, respectively).

In this post hoc analysis, the data were not pooled due to heterogeneity between the study populations. Despite this heterogeneity, bacterial eradication rates at 3–4 days occurred at high levels across all analyses. While limited by the inconsistency of repeat blood culture collection, the broad range of bacterial species seen in BSI and identified across the three reports indicate the efficacy of cefiderocol in both primary and select secondary BSI [52].

A prospective study by Falcone et al. builds on the investigation by Paterson and colleagues and evaluated the efficacy and safety of cefiderocol as a rescue therapy in 19 critically ill patients with serious infections (BSI or VAP) caused by carbapenem-resistant GNRs [53]. Complementing the findings of the post hoc analysis described above, these authors prospectively collected the data on 10 patients with ICU-acquired infections who were treated with cefiderocol. Patients were selected for treatment with cefiderocol if they had documented infections due to CR non-fermenting GNRs or CRE susceptible to cefiderocol and who had experienced clinical failure/and or severe adverse events from prior antibiotic regimens. All ten patients required mechanical ventilation, and six had a BSI (all A. baumanii). Clinical success occurred in 66.7% of cases. In a similarly small retrospective case series by Gatti et al., of thirteen patients treated with cefiderocol for management of XDR A. baumanii infections, eight had a BSI [54]. The 30-day mortality rate equaled 31%, and microbiological failure occurred in only one BSI patient who concurrently suffered from pneumonia.

In summary, the data describing the utility of and role for cefiderocol for BSI from resistant GNR are rather limited. This is not surprising given the short period for which it has been available. However, outcomes seem consistent with what one might predict given the severity of illness of the patients described and the pathogens encountered.

## 3. Conclusions

Multiple new agents exist for the treatment of severe MDR GNR infections. We have reviewed only several of the more commonly utilized agents, and thus, our report is somewhat limited. In general, though, they all have evidence supporting their role for a range of infections. However, assessing the proper place for these molecules in the treatment of BSI is complicated by the limited data describing their efficacy in this syndrome. Few patients in large RCTs for regulatory approval included subjects with concurrent BSI. Likewise, most analyses focusing on BSI specifically suffer from the limitations generally associated with all retrospective analyses. Despite these inadequacies, clinicians must make treatment choices when faced with a bacteremic patient. Thus, we have provided a summary of the relevant data and experience with these agents so that the decisions they make can be most informed.

## Data Availability

Not applicable.

## Appendix A

**Table A1:** Activity of select novel agents.

	Activity against Various Mechanisms of Resistance						
Agent	CRAB	ESBL	CRPA Non-MBL	CRE-CP	CRE-KPC	CRE-OXA-48	CRE-MBL
Ceftolozone–tazobactam	No	Yes	Yes	No	No	No	No
Ceftazidime–avibactam	No	Yes	Yes	+/−	Yes	Yes	No
Meropenem–vaborbactam	No	Yes	No	+/−	Yes	No	No
Cefidericol	Yes	Yes	Yes	Yes	Yes	Yes	Yes

Abbreviations: CRAB—carbapenem-resistant *A. bumanii*; CRE-CP—carbapenem-resistant *Enterobacteriaceae* carbapenemase producing; CRE-KPC—carbapenem-resistant *Enterobacteriaceae Klebsiella pneumoniae* carbapenemase; CRE-OXA-48—carbapenem-resistant *Enterobacteriaceae* OXA-48 enzyme producing, CRE-MBL—carbapenem-resistant *Enterobacteriaceae* metallo-beta-lactamase producer; ESBL—extended-spectrum beta-lactamase.
